# Comment on: Patellar instability in Indian population: relevance of tibial tuberosity and trochlear groove distance: SICOT J 2016, **2**, 14, DOI: 10.1051/sicotj/2016008

**DOI:** 10.1051/sicotj/2019018

**Published:** 2019-07-24

**Authors:** Balgovind S. Raja, Sai Gautham Balasubramanian

**Affiliations:** 1 Fellow in Arthroplasty, Fortis Escorts Hospital Delhi Gurgaon India; 2 Senior Registrar, KEM Hospital 400012 Mumbai India

Dear editor,

We were interested in reading “Patellar instability in Indian population: relevance of tibial tuberosity and trochlear groove distance” by Sourabh Kulkarni et al. published in the esteemed Sicot Journal Published online on 2016 Mar 25. DOI: 10.1051/sicotj/2016008 (PMID: 27163103). The objective of the study was to assess the TTTG distance in 100 MRI scans of skeletally matured Indian knees.

The authors defined TT–TG distance by measuring the distance between two parallel vertical lines, one passing through the apex of tibial tuberosity and the other through the apex of the trochlear groove [1]. The technique proposed in the study was based on Wittstein et al. [2] who recorded the TT–TG distance employing an axial image at the level of the insertion of the patellar tendon at the proximal tibial tubercle. But, the study used axial images at the level of the tendon distally for tuberosity reference. Insertion of the patellar tendon in tibia is crescent shaped [3]. Consequently, the measurement of the midpoint of the patellar tendon varies with each slice which in turn might skewer the final TT–TG values.

Furthermore, the author uses the center of the patellar tendon as a reference point for tibial tuberosity to obtain the TT–TG distance. This in fact is PT–TG (patellar tendon–trochlear groove) distance which is found to be different from the osseous TT–TG distance. Wilcox et al. [4] in his study revealed the variability in the insertion of the patellar tendon relative to the anterior aspect of the tibial tubercle and concluded that the TT–TG and PT–TG distance were not identical. In the present study the author had essentially used the PT–TG distance (center of the patellar tendon) instead of the anterior most point on the tibial tuberosity and assumed it to be TT–TG distance which might result in varied normal values in the population group.

To conclude, the methodology used in the study needs to be verified and the TT–TG distance measured should be ideally referred to as PT–TG distance.
